# Publisher’s Note: Continued Publication of *Audiology Research* by MDPI

**DOI:** 10.3390/audiolres10020008

**Published:** 2020-10-16

**Authors:** Shu-Kun Lin, Unai Vicario, Franck Vazquez

**Affiliations:** 1 MDPI, St. Alban-Anlage 66, CH-4052 Basel, Switzerland; lin@mdpi.com; 2 MDPI, Avenida Madrid, 95, 08028 Barcelona, Spain; vicario@mdpi.com

*Audiology Research* (ISSN 2039-4349) was launched in 2011 and published over the past nine years by PAGEPress Publications [1]. Dr. Gabriella Tognola served as the first Editor-in-Chief [2] and the second Editor-in-Chief, Professor Giacinto Asprella Libonati, is currently still active in this role. 

We are delighted to take over the publication of *Audiology Research* from PAGEPress and perpetuate the legacy of the journal. *Audiology Research* complements the portfolio of MDPI medical and pharmacology journals very well [3], and provides a medical complement to our existing physical journal *Acoustics* [4].

MDPI will publish the second issue of 2020 in this December, and thereafter publish four quarterly issues as of 2021. 

We are looking forward to publishing your research work in *Audiology Research* [5]!
